# Testing the Adaptive Significance of Personate Flowers in *Penstemon* (Plantaginaceae)

**DOI:** 10.1002/ece3.73692

**Published:** 2026-05-19

**Authors:** Trinity H. Depatie, Carolyn A. Wessinger

**Affiliations:** ^1^ Department of Biological Sciences University of South Carolina Columbia South Carolina USA

**Keywords:** floral adaptation, mating system evolution, *Penstemon*, personate flowers, pollinator specificity

## Abstract

Floral diversity across plant lineages reflects evolutionary shifts in pollinator specificity and mating strategy. In outcrossing species, floral traits may govern whether plants are visited by a generalized or specialized set of pollinators, while selfing is associated with traits that improve the ability to autonomously reproduce. *Penstemon*, a genus endemic to North America, exhibits striking floral diversity shaped by pollinator‐mediated selection. The genus contains three corolla morphologies—personate, tubular, and open‐tubed. Personate flowers are displayed in a handful of *Penstemon* species and are a peculiar bee‐pollinated floral type characterized by an upward bulge in the ventral portion of the corolla tube that obstructs the floral opening. Despite this flower morphology evolving independently a handful of times across the genus, little is known about the adaptive significance of personate flowers. To test the pollinator exclusion hypothesis, we observed bees visiting natural populations of *Penstemon* with open‐tubed, personate, and tubular flowers. We found that both large and small bees visit all three flower types, but small bees alter their visitation behavior to enter personate flowers. To evaluate the autonomous selfing hypothesis, we compared the mating systems of *P. hirsutus* (personate) and *P. smallii* (open‐tubed). Our mating system study revealed that *P. hirsutus* is more adapted to self‐fertilization than *P. smallii* , exhibiting reduced inbreeding depression and increased autonomous selfing success. Whether differences in self‐fertilization are due to flower shape differences is still unclear. Together, these findings attempt to contribute to understanding the evolution of floral diversity in *Penstemon* and demonstrate the adaptive significance of personate flowers.

## Graphical abstract

Floral diversity across plant lineages reflects evolutionary shifts in mating strategy and pollinator specificity. The genus *Penstemon* contains a handful of species that display personate flowers—a peculiar bee‐pollinated floral type characterized by an upward bulge in the ventral portion of the corolla tube that obstructs the floral opening. This study attempts to contribute to understanding the evolution of floral diversity in *Penstemon* and demonstrates that the adaptive significance of personate flowers is still murky.

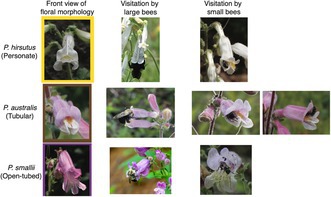

## 1 Introduction

Plant taxa display an extraordinary amount of floral trait diversity that reflects adaptive variation in mating system and pollination strategy. Plant mating strategies range from obligate outcrossing to primarily selfing, with a mixed mating strategy somewhere in between the extremes. Outcrossing requires a pollinating agent. Although a plant species may be able to produce viable seeds by self‐fertilization, outcrossing promotes genetic diversity and improves a population's ability to adapt (Barrett 2002). Selection favoring outcrossing has generated spectacular diversity in floral traits that attract, reward, and facilitate efficient pollination by the local pollinator community (Stebbins 1970; Ollerton et al. 2007). The abundance or effectiveness of specific pollinators will influence whether certain plant species evolve a generalist strategy by attracting a diverse assemblage of pollinators or a specialized strategy focused on few, but highly effective, pollinators (Armbruster 2017).

A generalist pollination strategy can be favored in environments with high levels of pollinator constancy and low amounts of competition among pollinators for resources (Waser et al. 1996; Ollerton 2017), leading to the evolution of flowers with open corollas, radial symmetry, and easily accessible rewards that attract a diverse group of pollinators (Faegri and van der Pijl 1979; Ollerton et al. 2007). However, there are circumstances where reliance on a particular pollinator species or functional type causes higher plant fitness. This scenario can lead to the evolution of floral traits that promote specialization and may even dissuade visitation by less effective pollinators (Grant and Grant 1967; Stebbins 1970; Johnson et al. 2006). For example, *Aquilegia*, *Linaria*, and *Tropaeolum* have all independently evolved a morphological key innovation, nectar spurs, that facilitates specialization through precise pollen deposition (Whittall and Hodges 2007; Fernández‐Mazuecos et al. 2019; Martínez‐Salazar et al. 2023). In each of these systems, adaptive variation in nectar spur length results in specialization on a pollinator type, such as lepidopterans, hummingbirds, or bees, by roughly matching the length of that pollinator's feeding structure. Flowers can also evolve morphological features that restrict access by certain types of pollinators to the rewards within. For example, hummingbird‐specialist *Penstemon* species have evolved narrow corolla tubes that restrict visitation by large bees (Castellanos et al. 2004). Plant‐pollinator specialization can improve a plant's reproductive efficiency and success in some circumstances, but relying on a single pollinator taxon or functional group in an unpredictable environment could be detrimental for plant fitness.

Despite the selective advantage of obligate outcrossing in flowering plants (Barrett and Cruzan 1994; Ferrer et al. 2025), mating system shifts to mixed mating or an obligate selfing strategy can be favored in environments with unpredictable pollinator abundance or scarcity of conspecifics (Baker 1955; Barrett 2002; Grossenbacher et al. 2015; Makowski et al. 2024). Mixed mating or self‐fertilization strategies provide plants with reproductive assurance in these conditions (Lloyd 1980; Schemske and Lande 1985). Due to their short window of time to reproduce, annual plants are perhaps more likely to benefit from the reproductive assurance provided by selfing or mixed mating compared to perennial plants that have multiple years to reproduce (Aarssen 2000). However, the evolution of selfing requires plants to evolve self‐compatibility (Fujii et al. 2016) and subsequently overcome the evolutionary hurdle of inbreeding depression by repeatedly purging deleterious recessive alleles from the population (Lande and Schemske 1985; Charlesworth and Charlesworth 1987; Eckert et al. 2006).

The evolution of selfing is typically accompanied by evolutionary shifts in floral traits to improve autonomous selfing rates and decrease investment in floral displays and rewards (Moeller and Geber 2005; Sicard and Lenhard 2011). Smaller flowers often lead to a reduction in the relative distance between male and female reproductive organs, facilitating autonomous selfing (Barrett 2002; Fishman and Willis 2008; Bodbyl Roels and Kelly 2011). For example, within *Capsella*, each shift to selfing is accompanied by the evolution of “selfing syndrome” traits such as reduced length and width of flower petals, decreased herkogamy, decreased pollen production, and increased ovule number (Slotte et al. 2012; Woźniak et al. 2020). Reduced pollen‐ovule (P:O) ratios reflect altered selection pressures on male versus female resource allocation in selfers that favor reduced investment in the production of pollen relative to ovules (Cruden 1977; E. L. Charnov 1979, 1982; Charlesworth and Charlesworth 1981).

*Penstemon* (Plantaginaceae) is a diverse genus of approximately 270 perennial wildflower species endemic to North America (Wolfe et al. 2006, 2021). *Penstemon* exhibits diversification in floral traits that reflects adaptation to distinct pollinator types (pollination syndromes), including repeated evolutionary shifts from flowers adapted to pollination by bees and wasps to those adapted to hummingbird pollinators (Wilson et al. 2007; Wessinger et al. 2019). Existing mating system studies in *Penstemon* have concluded that while *Penstemon* species are typically outcrossing, certain species are self‐compatible and can produce autonomously selfed seeds (Clements et al. 1999; Lange and Scott 1999; Lewinsohn and Tepedino 2007; Salas‐Arcos et al. 2017). Occasional selfing as part of a mixed‐mating strategy likely provides *Penstemon* plants reproductive assurance during years with infrequent pollinators.

Most *Penstemon* species displaying the bee pollination syndrome have relatively wide unobstructed corolla tubes and large lower petal lobes that form a landing platform for diverse bees and wasps—these traits have been experimentally shown to promote efficient pollination by several bee genera (Tepedino et al. 1999, 2007; Glenne 2003; Castellanos et al. 2004; Zung et al. 2015; Salas‐Arcos et al. 2019). Even *Penstemon* species that roughly conform to either bee or hummingbird syndrome may regularly receive other types of insect visitors, including flies, hawkmoths, and butterflies (Kimball 2008).

A rare floral type within *Penstemon* that is associated with bee pollination is the personate flower morphology—characterized by the presence of an upward bulge in the lower petal lobes that fully obstructs the floral passageway (Weberling 1992; Depatie and Wessinger 2025). This floral type evolved at least twice in the genus, leading to five personate‐flowered *Penstemon* species (Depatie and Wessinger 2025). The repeated evolution of personate flowers in *Penstemon* suggests they have been recurrently favored by selection; however, their ecological function is currently unknown. In this study we propose and test two hypothesized functions.

Our first hypothesis—the “exclusion hypothesis”—is that personate flowers in *Penstemon* prevent or discourage visitation by certain types of pollinators. Pennell (1935) proposed that only large bees manage to force their way into personate flowers; thus, the personate structure acts to exclude small bees. Crosswhite and Crosswhite (1966) argued instead that only small bees manage to squeeze into the crevices in the closed palate; thus, personate flowers act to exclude large bees. An exclusion hypothesis has also been proposed to explain the personate flowers of *Antirrhinum*, whereby personate flowers may function to reduce visitation by wasteful pollinators (Weberling 1992). We predict that only large or small bees, not both, would visit personate flowers, whereas bees of both sizes would visit open‐tubed flowers. A reduced range of insect visitor sizes effectively visiting personate relative to open‐tubed flowers would provide support for the exclusion hypothesis.

Our second hypothesis—the “autonomous selfing hypothesis”—posits that personate flowers in the genus increase the capacity for autonomous selfing. This hypothesis is based on our observation that the dorsoventral compression of the corolla tube in personate flowers decreases the vertical distance between floral reproductive organs, which may improve the likelihood of selfing in the absence of a pollinator visit. If personate‐flowered *P. hirsutus* (L.) Willd. is more adapted to self‐fertilization than open‐tubed *P. smallii* A. Heller, it may display additional traits associated with selfing, including reduced P:O ratios and reduced inbreeding depression.

In this study, we tested these two hypotheses using members of *Penstemon* section *Penstemon*. To test the exclusion hypothesis, we observed and identified insect visitors of several species with open‐tubed, personate, and tubular morphologies in natural populations to identify the size distribution of insect visitors. To test the autonomous selfing hypothesis, we conducted a controlled mating system study comparing two focal species with contrasting floral morphologies, *P. hirsutus* (personate) and *P. smallii* (open‐tubed).

## 2 Materials and Methods

### 2.1 Study System

**FIGURE 1:**
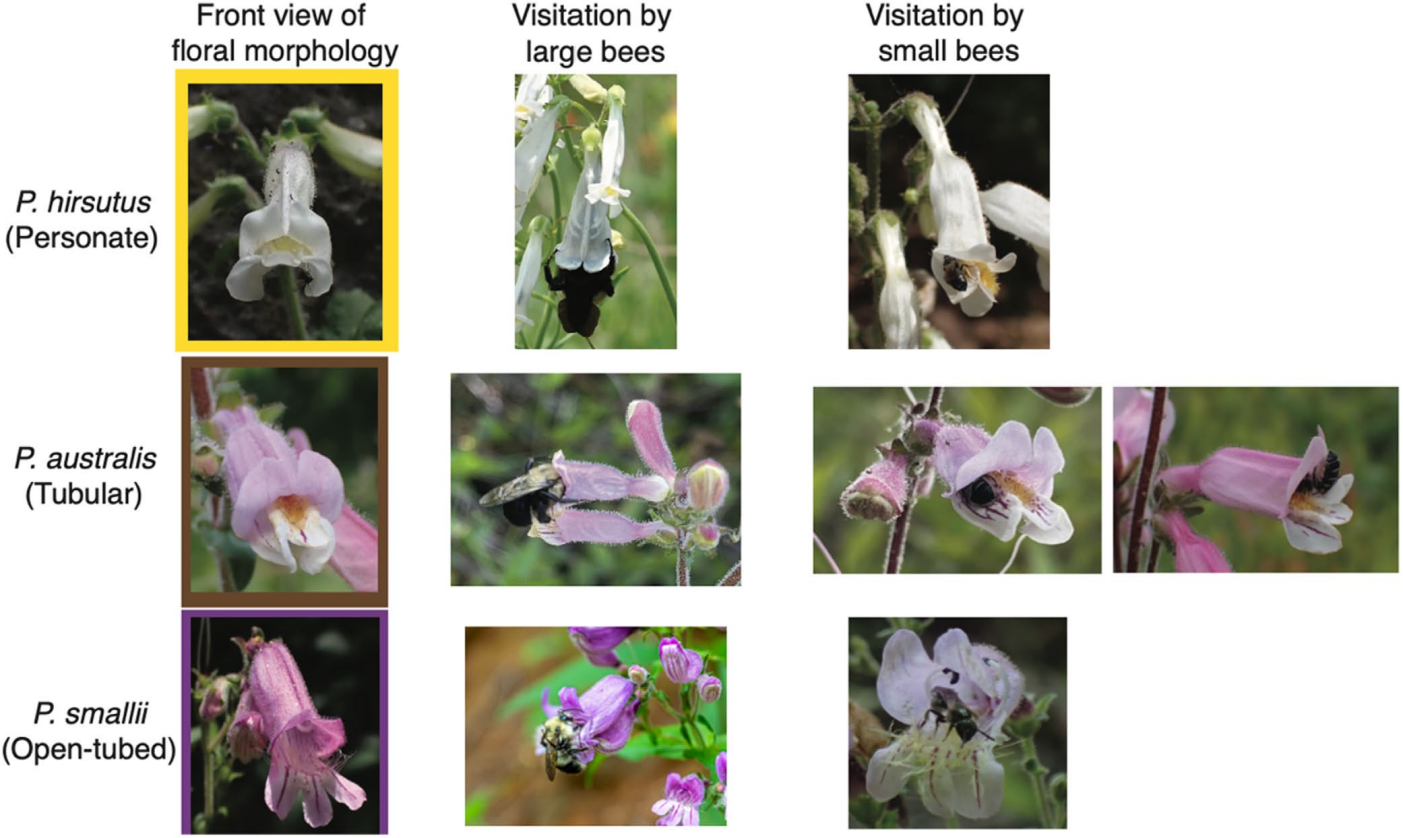
Representative floral morphology and bee visitation across *Penstemon* subsect. *Penstemon* species. Each row corresponds to a focal species and flower type. Photos depict differences in floral form and how bees of various sizes interact with each flower morphology during visitation. All images captured by THD.

Here we studied species of *Penstemon* subsection *Penstemon* found in the eastern half of North America, which display variation in corolla morphology (Figure 1). Within this clade, there are three species with personate flowers ( *P. hirsutus* , *P. oklahomensis* , and *P. tenuiflorus* ), three diploid species displaying open‐tubed (unobstructed) flowers, and six species with tubular flowers that are partially obstructed (Depatie and Wessinger 2025). We previously determined that ventral pleats—deep folds in the ventral petal tube tissue—are responsible for obstructing the floral opening and producing *Penstemon*'s personate flower morphology (Depatie and Wessinger 2025). *Penstemon* species with open‐tubed and tubular flowers still possess pleats on the ventral petal surface, but they are not nearly as deep or obstructive as the pleats in personate flowered species (Figure 1). Moreover, phylogenomic analyses with whole‐genome resequencing data revealed two evolutionary origins of the personate floral type within *Penstemon* subsect. *Penstemon*, suggesting it has repeatedly been favored by selection (Depatie and Wessinger 2025).

### 2.2 Floral Nectar Collection

To understand how floral nectar volume varies across focal *Penstemon* subsect. *Penstemon* species, we quantified floral nectar production of plants grown in temperature‐controlled growth chambers at the University of South Carolina (additional methods in plant propagation, below). Nectary type—modified glandular trichomes on the surface of two lateral stamen filaments—is conserved across *Penstemon*; however, nectar volume and nectary area are evolutionarily labile across pollination syndromes (Katzer et al. 2019, 2025). At a consistent time (10 am) on the first day of flower opening, we used 2 μL micro‐capillary tubes to collect and measure nectar volume. We measured two to four flowers on 15 plants across the following species: *P. canescens* Britton, *P. hirsutus* , *P. laevigatus* Aiton, *P. laxiflorus* Pennell, *P. smallii* , *P. tenuiflorus* Pennell, and *P. tenuis* Small.

### 2.3 Insect Visitor Observations

We observed insect visitors during the spring and summer of 2022 (April through June) in natural populations of seven focal *Penstemon* species (Table 1). We selected populations for observations that contained at least 20 plants at a similar flowering stage, where an observer could view several flowering stalks at once. We performed observations for a total of 35.5 h across all populations, divided into 30‐min observation periods. During each observation period, each observer closely watched a group of 10–15 plants, and all bees that legitimately visited a flower were captured in separate 25 mL Eppendorf snap‐cap tubes. We considered a flower visit to be legitimate once a bee visibly entered the part of the flower where the reproductive organs are located. We submerged the 25 mL snap‐cap tube holding the bee in a cooler with ice to slow its metabolism. To ensure that bees were not being released and then recaptured minutes later, we kept bees on ice until the end of all observation periods each day. We then individually removed the chilled bees from their 25 mL tube, photographed each bee for identification, and measured their thorax height with digital calipers. Following measuring and imaging, we placed bees in an open container and waited for them to wake up and fly off.

**TABLE 1:** Focal *Penstemon* species and the duration of pollinator observations conducted for each species.

*Penstemon* species	Floral type	Number of plant populations visited	Total observation time	Plants collected for mating system study
*P. australis*	Tubular	2	7.5 h	No
*P. canescens*	Tubular	1	3 h	No
*P. hirsutus*	Personate	1	6 h	6 plants
*P. oklahomensis*	Personate	1	6 h	No
*P. tenuiflorus*	Personate	1	3 h	No
*P. calycosus*	Open‐tubed	1	6 h	No
*P. smallii*	Open‐tubed	1	4 h	3 plants

### 2.4 Analysis of Visitation Data

We used the photographs of bees taken in the field and identification books (Williams et al. 2014; Wilson and Carril 2016) to determine the genus‐level identity of each bee visitor. To determine whether the distribution of visitor size depends on flower morphology, we used the lm function of the *stats* package in R (v4.3.0; R Core Team 2023) to fit a linear model with insect height as the response variable and flower shape (open‐tubed vs. personate vs. tubular) as the predictor variable.

### 2.5 Plant Propagation

We chose personate‐flowered *P. hirsutus* and open‐tubed *P. smallii* as the focus of a comparative mating system study because these species represent the opposing extremes of flower shape variation in *Penstemon* subsect. *Penstemon* (Depatie and Wessinger 2025). We grew plants obtained from a native plant nursery (Wood Thrush Native Nursery, Floyd, Virginia, USA) and from natural populations (see Table 1) in temperature‐controlled growth chambers at the University of South Carolina under 18 h of daylength and temperatures ranging from 18°C at night to 24°C during the day. To induce flowering, we vernalized plants for 8 weeks at 4°C. Following vernalization, we placed the plants under broad spectrum lights and fertilized with Jack's Classic Blossom Booster (JR Peters Inc., Allentown, Pennsylvania, USA).

### 2.6 Quantifying Pollen‐Ovule Ratios in *P. hirsutus* and *P. smallii*

We compared pollen‐ovule (P:O) ratios between *P. hirsutus* and *P. smallii* . Following Jürgens et al. (2002), we collected three flower buds from three *P. hirsutus* and three *P. smallii* plants (*n* = nine buds per species) one day prior to flower opening. We preserved individual flower buds in 2 mL Eppendorf tubes with 70% ethyl alcohol and stored them at 4°C. We individually placed two anthers per flower into 0.6 mL tubes with 15 μL of water and vortexed to release pollen. We transferred the pollen solution to a gridded slide and photographed the pollen using a Leica Stereo Microscope equipped with a Flexacam C3 (Wetzlar, Germany). We dissected ovules from the ovary on a watch glass in a pool of lactic acid to help separate the ovules, following methods reported in Baur et al. (2021). We photographed the floating ovules using a digital microscope (DinoLite, Torrance, California, USA) attached to a desktop computer. We used the Cell Counter plugin in Fiji (Schindelin et al. 2012) to assist with manual counting of pollen grains and ovules. We multiplied the average number of pollen grains per anther by four to calculate the number of pollen grains per flower. We calculated per‐flower P:O ratios by dividing the number of pollen grains per flower by the number of ovules per flower (Cruden 1977). We compared P:O ratios between *P. hirsutus* and *P. smallii* with two sample *t*‐tests using the t.test function of the *stats* package in R.

### 2.7 Mating System Study

**FIGURE 2:**
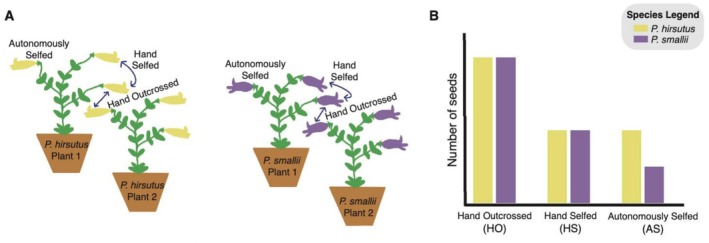
Experimental design for the mating system study. (A) Illustration of the three pollination treatments conducted with personate and open‐tubed flowers to determine each species' ability to produce hand outcrossed (HO), hand selfed (HS), and autonomously selfed (AS) seeds. (B) Hypothesized results that would suggest *P. hirsutus* flowers are better adapted for autonomous selfing.

We compared the mating system of *P. hirsutus* and *P. smallii* using controlled crosses conducted in pollinator‐free growth chambers. For each species, we performed three pollination treatments: hand outcrossed (HO) using pollen from a different randomly‐chosen conspecific individual, hand selfed (HS) using pollen from another flower of the same individual, and autonomous selfed (AS) where flowers were left unmanipulated (Figure 2). To prevent unintended self‐pollination, we removed the anthers assigned to the HO and HS treatment groups prior to their dehiscence. We conducted pollinations for the HO and HS treatments by applying ample amounts of pollen to receptive stigmas—typically 2 days following flower opening. We collected all capsules at the time of ripening and stored them in paper coin envelopes on silica gel at room temperature for one month after collection. We then counted and weighed seeds per capsule and recorded capsules that contained zero seeds.

We made two comparisons in our seed set data. First, we compared the relative seed set in the HS to the HO treatment to assess the severity of inbreeding depression—a lower HS:HO ratio indicates greater early acting inbreeding depression. Second, we compared the seed set in the AS to the HS treatment to assess the capacity of autonomous self‐pollination—a higher AS:HS ratio indicates a greater ability to autonomously self. We tested the hypothesis that *P. hirsutus* has a superior ability to autonomously self‐fertilize relative to *P. smallii* (Figure 2). Some plants were missing certain treatment groups (Appendix S1), therefore, to compare seed set ratios we only used seed count data from plants that produced seeds in both focal treatments (HS and HO or AS and HS). Before calculating the ratios, we divided the number of seeds produced per plant in each treatment group by the number of capsules to account for differences in seed number due to overall plant reproductive effort.

### 2.8 Mating System Modeling Approach

To analyze the seed production data, we fit a hurdle generalized linear mixed model using the *glmmTMB* package in R (McGillycuddy et al. 2025; Brooks et al. 2017). Hurdle models are two‐part models that separately estimate the probability of a nonzero outcome and the conditional distribution of positive counts. This model allowed us to distinguish between (1) the binary process of whether a capsule succeeded or failed to produce any seeds and (2) variation in seed number per capsule for successful pollination events. First, the binary (zero hurdle) component modeled the probability of seed production as a function of species, mating system, and their interaction. Next, the count component modeled the number of seeds produced per capsule using a truncated Poisson distribution, which excludes zeros by design, with the same fixed effects as the binary component. Results from both model components were used to assess the effects of species identity and pollination treatment on the likelihood of seed set and on seed output when successful. We were unable to include plant as a random effect in our hurdle model due to low levels of replication of our plant and treatment combinations (Appendix S1).

We also compared seeds per capsule, average seed mass, and failure to set seeds using linear mixed effects models using the glmer function of the *lme4* package in R (Bates et al. 2015), including maternal plant as a random effect to account for the genetic background. Within these linear mixed effects models, we included an interaction between the fixed effects (species and pollination treatment). We obtained estimated marginal means (EMMs) with the emmeans function of the *emmeans* package in R (Lenth 2025), which estimates the pairwise differences between the means of each focal group in the linear mixed effects models. For models comparing three or more focal groups, we conducted an emmeans *p* value adjustment via the tukey method. Finally, we fit linear mixed effects models for HS:HO and AS:HS treatment comparisons across maternal plants.

## 3 Results

### 3.1 Nectar Volume Varies Across *Penstemon* Subsect. *Penstemon* Species

**FIGURE 3:**
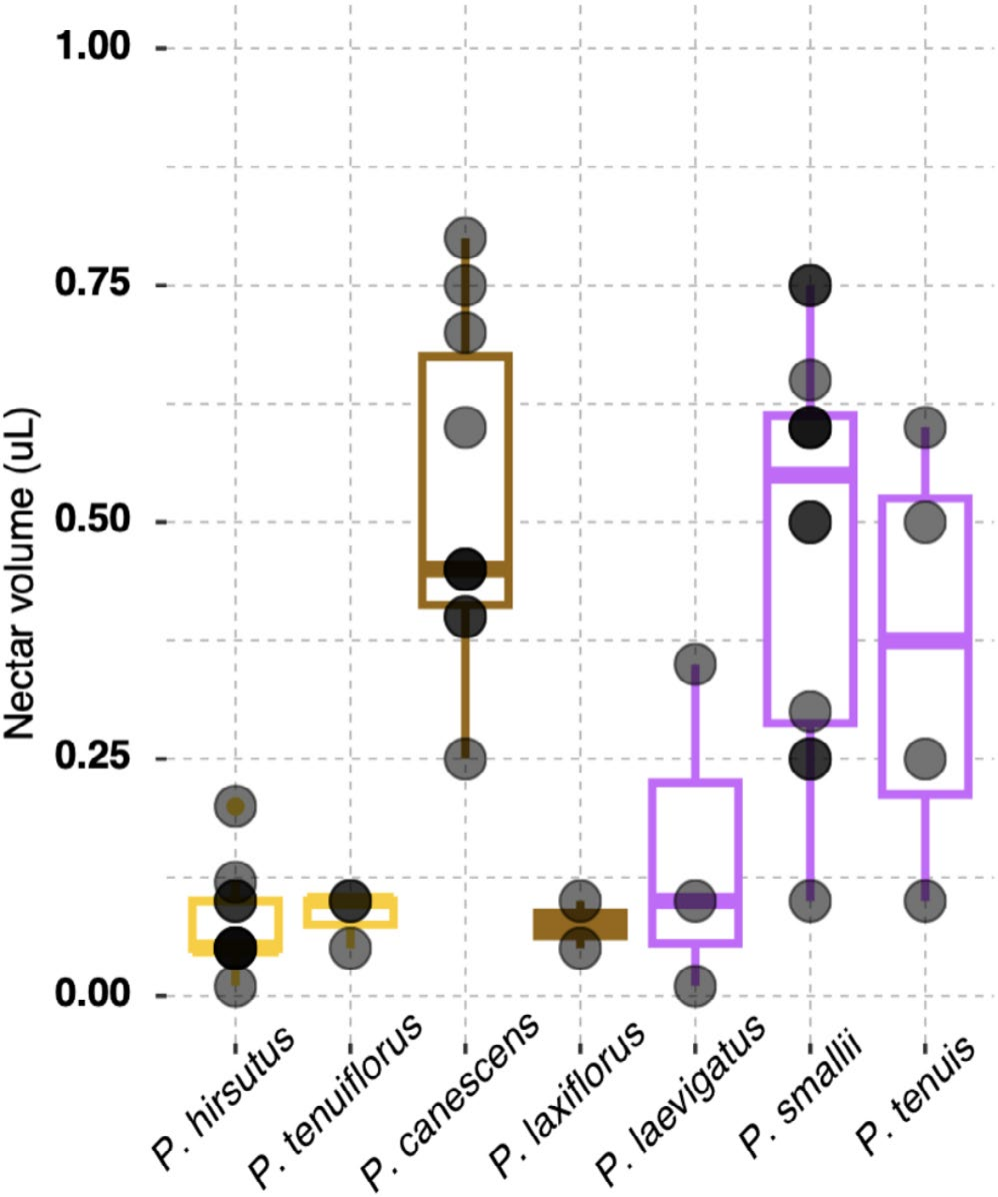
Nectar volume measurements from various *Penstemon* subsect. *Penstemon* species. Each black point represents the volume of nectar produced in a newly opened flower. The outline color of each box matches flower shape (yellow: Personate, brown: Tubular, purple: Open‐tubed) and box lines indicate the median volume of nectar produced per species.

Nectar production was consistently low in personate‐flowered species ( *Penstemon hirsutus* and *P. tenuiflorus* ; Figure 3). Median nectar volume differed across floral morphologies, where tubular and open‐tubed flowers typically produced more nectar than personate flowers; however, within species variability was prevalent (Figure 3).

### 3.2 Small Bees Show Different Behaviors When Visiting Personate and Open‐Tubed Flowers

Interestingly, we found a noticeable difference in how large versus small bees enter and exit personate and tubular flowers (Figure 1). Large bees approach personate and tubular flowers upright and grab onto the closed floral petals with their front two legs to pry the flower open. When exiting these flowers, the large bees simply back their head out of the flower, release the petals from the grasp of their forearms, and fly off in the same upright orientation that they entered. Small bees land on one of the two external ventral petals of personate and tubular flowers and squeeze their way through a tiny opening between the top and bottom flower petals. Small bees exit the morphologically complex flowers upside down and through the middle of the flower (Figure 1) so that their head exits the flower last and their wings are pointed down towards the lower floral lip. By contrast, both large and small bees visit open‐tubed flowers using a similar approach: bees of both sizes land on the flower's extended lower petal lobes and crawl into the flower to gather reward. Both bee size classes leave open‐tubed flowers by backing out of the corolla tube and then flying off to another flower.

### 3.3 Personate Flowers Do Not Appear to Filter Bees Based on Their Size

**FIGURE 4:**
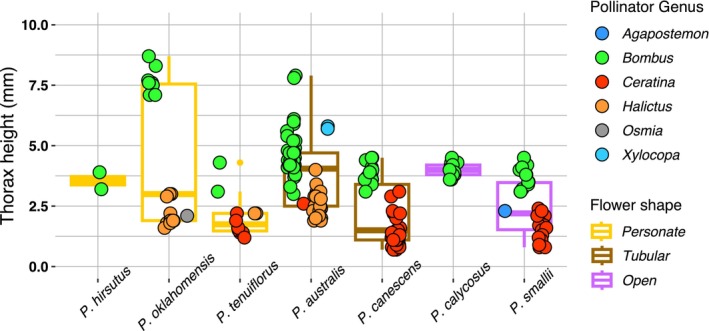
Floral visitor thorax height was variable across focal *Penstemon* subsect. *Penstemon*'s species. Each point represents a bee that legitimately visited a flower. Point color corresponds with the genus of the bee visitor, and the outline color of each box matches flower shape (brown: Tubular, purple: Open‐tubed, yellow: Personate). Box lines indicate the median thorax height of bees that visited each flower species.

We observed a total of 185 legitimate bee visits across all focal *Penstemon* species. Personate‐flowered species ( *P. hirsutus* , *P. oklahomensis* , and *P. tenuiflorus* ) received 34 legitimate visits, tubular‐flowered species ( *P. australis* and *P. canescens* ) received 108 visits, and open‐tubed species ( *P. smallii* and *P. calycosus* ) received 43 visits (Figure 4). We found no statistical difference in thorax height of floral visitors between each of the three flower types (*F*‐statistic = 1.391; *p* value = 0.2515; Appendix S2).

### 3.4 *P. hirsutus* and *P. smallii* Exhibit Similar Pollen‐Ovule Ratios

**FIGURE 5:**
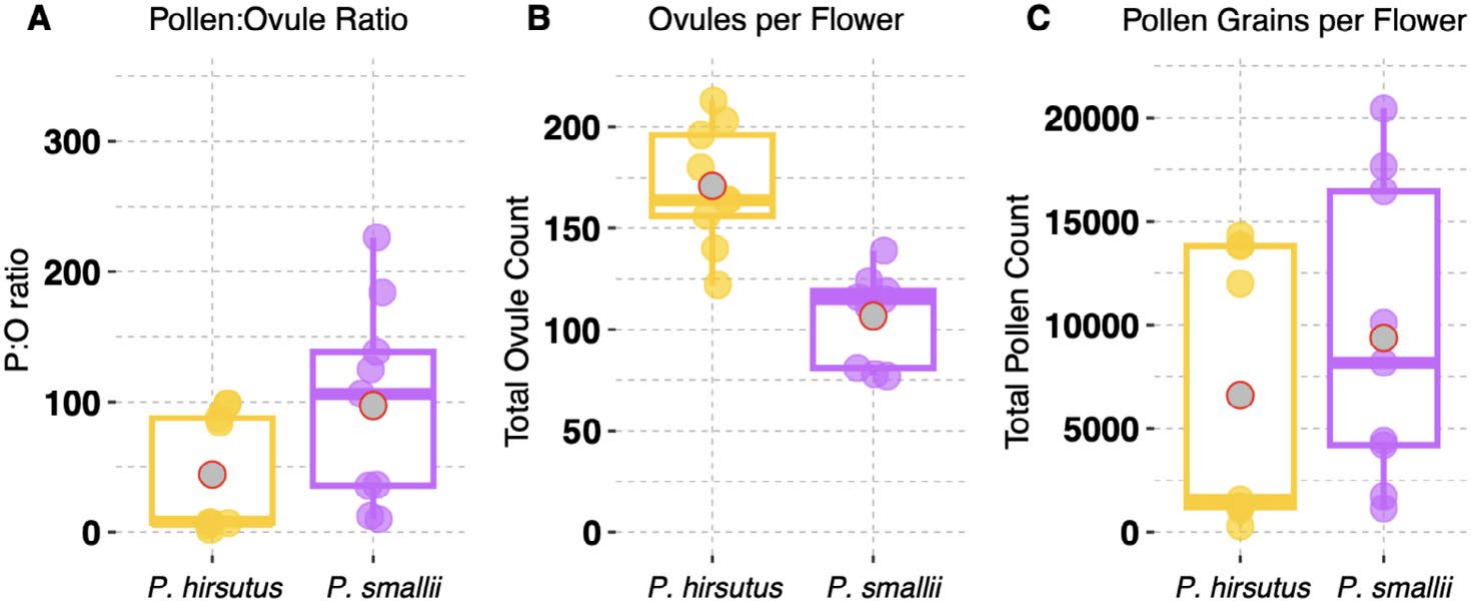
Comparisons of pollen grains and ovule counts between *P. hirsutus* and *P. smallii* . (A) Pollen‐ovule ratios. (B) Ovule counts. (C) Pollen counts. Colored points represent the value that corresponds with each flower in the dataset (*n* = 9 per species) and gray points with a red outline indicate the mean value for each species.

We quantified pollen‐ovule (P:O) ratios between personate *P. hirsutus* and open‐tubed *P. smallii* to examine whether personate flowers are associated with a reduced male reproductive investment and/or an increased female investment. P:O ratios were variable among flowers within an individual plant and not significantly different between the two species (*p* value = 0.2384; Figure 5A; Appendix S3). *P. hirsutus* produces significantly more ovules than *P. smallii* (*p* value = 0.03365; Figure 5B; Appendix S4) and similar, though highly variable, amounts of pollen (*p* value = 0.5228; Figure 5C; Appendix S5).

### 3.5 Across Treatment Groups *P. hirsutus* Produces More Seeds Per Capsule Than *P. smallii*

**FIGURE 6:**
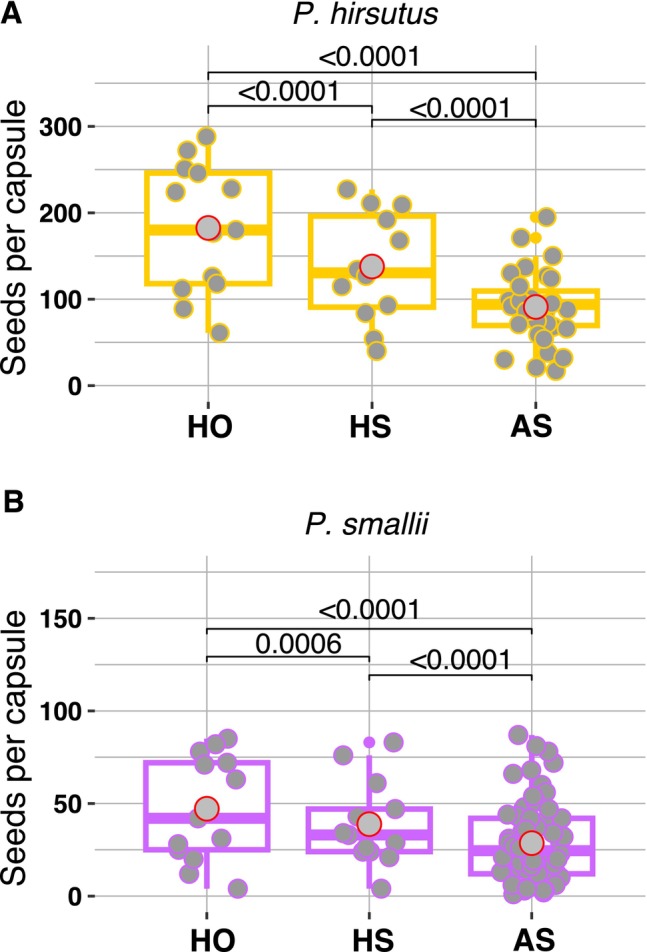
The total number of seeds per capsule produced by (A) *P. hirsutus* , and (B) *P. smallii* across all three treatment groups. Each point represents the number of seeds per capsule. Box lines indicate the median and gray points with a red outline signify the mean number of seeds per treatment group. Statistical significance between treatment groups within each species was assessed with a linear mixed effects model and *p* values are printed above lines displaying the respective comparison.

**FIGURE 7:**
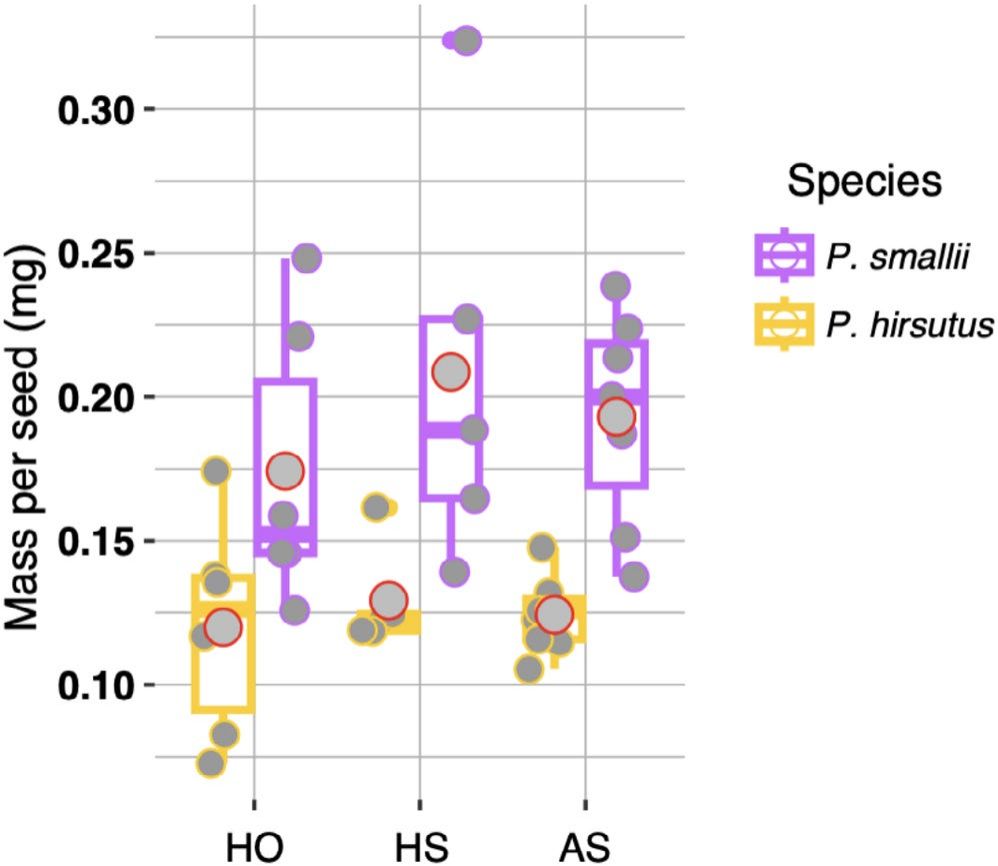
The mass in milligrams (mg) for *P. hirsutus* (yellow) and *P. smallii* (purple) seeds. Each point represents the average seed mass per plant/treatment combination. Box lines indicate the median and gray points with a red outline signify the mean seed mass value for each species/treatment combination.

Both *P. hirsutus* and *P. smallii* set seeds in the selfed treatments, indicating that both species are self‐compatible. Regardless of pollination treatment, *P. hirsutus* produced many more seeds than *P. smallii* (*p* value = 0.00271; Figure 6A; Appendix S6). This result is expected because *P. hirsutus* produces significantly more ovules than *P. smallii* (Figure 5B). Each species produced significantly fewer seeds in the HS treatment relative to HO as well as significantly fewer seeds in the AS treatment relative to the HS treatment (Figure 6; Appendix S7). We found no overall significant difference between the mass of *P. hirsutus* and *P. smallii* seeds (*p* value = 0.189; Figure 7; Appendix S8).

### 3.6 Personate‐Flowered *P. hirsutus* Is More Reliable at Producing Self‐Fertilized Seeds Than *P. smallii*

**FIGURE 8:**
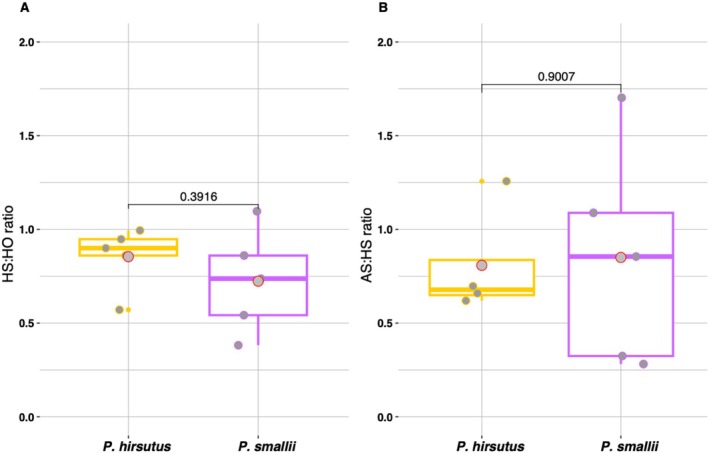
Pairwise comparisons between seed set in the (A) hand selfed and hand outcrossed treatment groups (HS:HO) and the (B) autonomously selfed and hand selfed treatment groups (AS:HS). Points represent data for a given plant. Box lines indicate the median and gray points with a red outline signify the mean ratio value for each species. Statistical significance between species within each ratio comparison was assessed with a linear mixed effects model and *p* values are printed above lines displaying the comparison.

*P. hirsutus* and *P. smallii* both set significantly fewer seeds in the HS treatment compared to the HO treatment (Figure 6), suggesting that both species are suffering from deleterious effects of inbreeding depression. Even though the difference between species' HS:HO treatment comparison is not significant (F‐statistic = 0.8203, *p* value = 0.3916; Figure 8A; Appendix S9), it is notable that across multiple plants *P. hirsutus* had greater HS:HO ratios than *P. smallii* . Furthermore, *P. smallii* failed to produce capsules with seeds in 31.6% of HS capsules while *P. hirsutus* only failed to set seeds in the AS treatment (Table 2; Appendices S10 and S11). Taken together, these results suggest that *P. smallii* is suffering from stronger effects of early‐acting inbreeding depression than *P. hirsutus* . Alternatively, *P. smallii* could have a reduced degree of self‐compatibility compared to *P. hirsutus* .

**TABLE 2:** The number of capsules produced with and without seeds in each treatment group for *P. hirsutus* and *P. smallii* .

Mating system	*Penstemon* species	Number of capsules with seeds	Number of capsules without seeds	Rate of seed set failure (%)
Hand outcrossed	*P. hirsutus*	13	0	0.0
Hand outcrossed	*P. smallii*	13	1	7.14
Hand selfed	*P. hirsutus*	12	0	0.0
Hand selfed	*P. smallii*	13	6	31.58
Autonomously selfed	*P. hirsutus*	35	3	7.89
Autonomously selfed	*P. smallii*	70	38	35.19

*Note:* Species rate of seed failure was calculated by dividing the number of capsules without seeds by the number of capsules with seeds for each species and treatment group combination.

Both species produced fewer seeds in the AS treatment compared to the HS treatment (Figure 6) and both also failed to set seeds at a higher rate in the AS treatment relative to the HS treatment— *P. smallii* failed to set seeds in 35.2% of AS capsules while the failure rate for *P. hirsutus* was 7.9%. However, the two species did not differ significantly in AS:HS treatment comparisons for capsules that successfully produced seeds (*F*‐statistic = 0.01674, *p* value = 0.9007; Figure 8B; Appendix S12). Therefore, based on AS:HS ratios alone, we see no clear evidence that *P. hirsutus* has a higher capacity for autonomous selfing relative to *P. smallii* .

## 4 Discussion

### 4.1 The Insect Visitors of Personate Flowers in *Penstemon*

Despite prior speculation that personate *Penstemon* flowers filter pollinators by body size (Pennell 1935; Crosswhite and Crosswhite 1966), we found that personate‐flowered *Penstemon* species are visited by both large and small bees. Our results are consistent with observations by Clements et al. (1999) who found that natural populations of the personate species *P. hirsutus* and *P. tenuiflorus* are visited by bees of various sizes. Our observations did reveal an interesting difference in how small and large bees handle personate *Penstemon* flowers compared to open‐tubed flowers. Specifically, the upside‐down pollination behavior of small bees in personate flowers may alter their visitation duration, which could have effects on pollination efficiency. Like the current study, A. J. Beattie (1971, 1974) found evidence of both nototribic (upright) and sternotribic (upside down) pollinators in *Viola*. *Viola rostrata* and *V. blanda* have overlapping ranges and have diverged in reproductive organ arrangements facilitating pollination by these two pollinator types—successful pollination in *V. rostrata* occurs almost exclusively by nototribic bees, whereas *V. blanda* is most effectively pollinated by sternotribic bees (A. J. Beattie 1974). This work suggests that certain *Viola* species have evolved floral traits that favor outcrossing via a specialized pollinator type. The findings of our *Penstemon* visitation study indicate that personate flowers are visited by both large and small bees and that small bees adjust their visitation behavior to gain access to the reward in the morphologically complex personate and tubular flowers. However, because our experiment does not investigate potential pollination efficiency differences between large and small bee visitors, we do not currently have enough evidence to determine whether personate flowers are specialized to a particular bee size. Future ecological studies to quantify whether the observed behavioral difference impacts relative pollination efficiency are needed.

### 4.2 Personate *Penstemon* Species Compete for Floral Visitors in Environments With Showy‐Flowered Species

Both *P. hirsutus* (personate) and *P. calycosus* (open‐tubed) occurred at one of our observation sites, and competition between flowering species may have reduced the pollinator visitation rate to *P. hirsutus* . Bees appeared to preferentially visit *P. calycosus* over *P. hirsutus*; however, bees mostly appeared to prefer visiting the other flowering plants that co‐occurred at the site, including Indigo Bush ( *Amorpha fruticosa* ), Widow's Cross ( *Sedum pulchellum* ), and the Purple Spiderwort ( *Tradescantia virginiana* ). The data we collected in this experiment provide an opportunity to compare visitation rates between the species and morphological groups (Appendices S13 and S14). However, habitat differences between focal *Penstemon* species, flowering phenology, insect and co‐flowering plant communities, and the temporary removal of bees in our experimental design complicate this comparison (Moeller and Geber 2005; Lázaro et al. 2013; Bennett and Lovell 2019; Ruppel et al. 2025).

Consistent with the notion that perhaps bee visitors find the morphologically complex personate flowers less desirable and less attractive than the open‐tubed and tubular *Penstemon* flowers, the personate species also display pale white flowers that lack nectar guides and produce very small volumes of nectar. This suite of traits associated with personate flowers in *Penstemon* subsect. *Penstemon* makes it difficult to pinpoint which flower trait might drive any differences in insect visitation. Manipulative experiments that determine how individual trait differences contribute to pollinator response are required to disentangle integrated floral features that influence pollinator visitation (e.g., Meléndez‐Ackerman et al. 1997, Meléndez‐Ackerman and Campbell 1998; Waser and Price 1983, 1985). For example, Salas‐Arcos et al. (2019) manipulated several morphological traits that are often associated with pollinator divergence like flower angle and flower width in field populations of *Penstemon gentianoides* and found that a reduction in both traits caused a decrease in bumblebee foraging and pollination success. Alternatively, the effects of floral traits on pollinator visitation could be disentangled using recombinant hybrid populations (e.g., Schemske and Bradshaw Jr 1999).

### 4.3 Comparative Mating System Study Provides Evidence That *P. hirsutus* Is More Adapted to Self‐Fertilization Than *P. smallii*

Our mating system experiment confirmed that *P. hirsutus* is self‐compatible (Clements et al. 1999; Ksiazek‐Mikenas et al. 2019) and presents new data revealing that *P. smallii* is also self‐compatible. In fact, self‐compatibility has been identified in several other *Penstemon* species such as *P. digitalis* , *P. scariosus* var. *albifluvis* , *P. bicolor* , and *P. pinifolius* (Zorn‐Arnold and Howe 2007; Lewinsohn and Tepedino 2007; Glenne 2003; Lange et al. 2000, respectively). Despite being self‐compatible, both *P. hirsutus* and *P. smallii* had reduced seed production in the hand selfed treatment relative to hand outcrossed, suggesting early acting inbreeding depression associated with selfing (Husband and Schemske 1996; Brandvain et al. 2024).

Despite identifying significant differences between the number of seeds produced per treatment group for each species, we did not detect a significant difference between the ratio of seeds produced in either the HS:HO or AS:HS treatment comparisons. We did, however, find that *P. hirsutus* is a more reliable selfer—by failing to produce HS seeds less frequently—and that it has a slightly lower P:O ratio than *P. smallii* . Taken together, these lines of evidence suggest that personate‐flowered *P. hirsutus* may be better adapted to self‐fertilization than open‐tubed *P. smallii* . A mating system study involving many more plants would help identify a more subtle difference in selfing capacity. An increased capacity for self‐pollination would not necessarily mean that *P. hirsutus* is evolving towards becoming an obligate selfing species—it could signal a mixed‐mating strategy for reproductive assurance when ecological circumstances are unfavorable. Perennials, like *Penstemon* wildflowers, are predicted to experience weaker selective pressures to evolve selfing for reproductive assurance compared to annuals that have only one season to reproduce (Aarssen 2000).

### 4.4 Differences in the Rate of Seed Set Failure Between *P. hirsutus* and *P. smallii* do Not Appear to be Related to Flower Morphology

Our reproductive assurance hypothesis for the evolution of the personate flower shape predicted that *P. hirsutus* would have a lower proportional reduction in seeds set for the autonomous self‐pollination treatment compared to *P. smallii* . However, our data do not support this prediction: *P. hirsutus* experienced an ~8% decline in successful seed set in autonomous versus hand self‐pollination, while the corresponding rate of decline for *P. smallii* was only ~5%. Instead, our data mainly reveal that *P. smallii* flowers suffered a similarly high seed set failure rate compared to *P. hirsutus* in both hand and autonomous self‐pollination treatments, implicating higher inbreeding depression affecting *P. smallii* . In other words, the greater selfing capability of *P. hirsutus* compared to *P. smallii* may be entirely explained by reduced early acting inbreeding depression rather than an improved ability to autonomously self.

### 4.5 Caveats of Our Mating System Study

While our mating system study compared the levels of self‐compatibility, early acting inbreeding depression, and autonomous selfing capabilities between the two focal *Penstemon* species, it did not assess later‐acting forms of inbreeding depression such as the viability of self‐fertilized seeds and the fitness of self‐fertilized offspring. Germinating the seeds from this mating system study would inform on whether selfed progeny experience reduced survival. For example, selfed progeny of *Impatiens capensis* experienced lower overwinter survivorship and a growth disadvantage (Mitchell‐Olds and Waller 1985). Yet there are examples where selfed progeny do not exhibit reduced fitness, like the high survival and fecundity of selfed *Gilia achilleifolia* (Schoen 1983).

## 5 Conclusion

Personate flowers in *Penstemon* subsect. *Penstemon* do not appear to filter insect visitors by size, but we did find a clear difference in the way that small bees visit morphologically complex personate and tubular flowers. Because we did not quantify the rate of pollen transfer in this experiment, we do not know whether any observed visitors are efficient pollinators or are acting as thieves. Future work should quantify visitation by collecting pollen transfer data and consider transplanting focal species to a single environment and pollinator community. Together these data would provide more evidence to definitively say whether personate flowers are specialized to receive effective visits from a single bee size class. Contrary to the reproductive assurance hypothesis, personate flowers do not seem to directly influence the capacity for autonomous selfing. Our focal personate‐flowered species— *P. hirsutus* —does possess features associated with a greater capacity for selfing, including reduced inbreeding depression, lack of floral pigmentation, and reduction in floral nectar production. It is therefore possible that personate flowers do reflect a shift in mating system; however, our modest‐sized experiment was unable to clearly affirm or refute this hypothesis. Even though both focal species are capable of autonomous selfing, the mating system of *P. hirsutus* and *P. smallii* should be investigated in natural populations. The ecological function of personate flowers in *Penstemon* remains enigmatic but deserves further attention.

## Author Contributions

**Carolyn A. Wessinger:** conceptualization (equal), funding acquisition (lead), methodology (equal), project administration (lead), supervision (lead), writing – original draft (supporting), writing – review and editing (supporting). **Trinity H. Depatie:** conceptualization (equal), data curation (lead), formal analysis (lead), methodology (equal), visualization (lead), writing – original draft (lead), writing – review and editing (lead).

## Funding

This work was supported by the National Science Foundation, DEB‐2052904. National Institutes of Health (NIH), R35GM142636.

## Conflicts of Interest

The authors declare no conflicts of interest.

## Supporting information


**Appendix S1:** Number of *P. hirsutus* and *P. smallii* plants used for the mating system study and for specific treatment group comparisons.
**Appendix S2:** Linear model results when investigating whether flower shape explains the variation in pollinator thorax height.
**Appendix S3:** Two sample t‐test used to investigate whether species explains the variance in pollen‐ovule ratios.
**Appendix S4:** Linear model results when investigating whether species explains the variance in ovule number.
**Appendix S5:** Linear model results when investigating whether species explains the variance in pollen grains per flower.
**Appendix S6:** Hurdle model results when investigating the variance in seed production and number per capsule.
**Appendix S7:** Estimated Marginal Means compared between treatment groups for both species' seed number.
**Appendix S8:** Linear model results when investigating whether an interaction between species and mating system (fixed effects) explains the variance in seed mass.
**Appendix S9:** Linear model results when investigating whether species explains the variance in the HS:HO ratio.
**Appendix S10:** Linear model results when investigating whether an interaction between species and mating system (fixed effects) explains the variance in the rate of seed failure.
**Appendix S11:** Comparisons of capsule production with and without seeds in the hand outcrossed (HO), hand selfed (HS), and autonomously selfed (AS) mating systems for *P. hirsutus* and *P. smallii* .
**Appendix S12:** Linear model results when investigating whether species explains the variance in the AS:HS ratio.
**Appendix S13:** Hourly visitation rate for each plant species per observation day.
**Appendix S14:** Hourly visitation rate per day by bees differs across focal plant populations.

## Data Availability

All data and R scripts that support this research are deposited in a Zenodo repository: https://doi.org/10.5281/zenodo.18089937.
