# Supplementary material for: Testing the Adaptive Significance of Personate Flowers in Penstemon (Plantaginaceae)
Source: Ecol Evol. 2026 May 19;16(5):e73692. doi: 10.1002/ece3.73692 (PMC13185559; doi:10.1002/ece3.73692)
Supplement: Supplementary file 1 — Appendix S1: Number of P. hirsutus and P. smallii plants used for the mating system study and for specific treatment group comparisons. Appendix S2: Linear model results when investigating whether flower shape explains the variation in pollinator thorax height. Appendix S3: Two sample t‐test used to investigate whether species explains the variance in pollen‐ovule ratios. Appendix S4: Linear model results when investigating whether species explains the variance in ovule number. Appendix S5: Linear model results when investigating whether species explains the variance in pollen grains per flower. Appendix S6: Hurdle model results when investigating the variance in seed production and number per capsule. Appendix S7: Estimated Marginal Means compared between treatment groups for both species' seed number. Appendix S8: Linear model results when investigating whether an interaction between species and mating system (fixed effects) explains the variance in seed mass. Appendix S9: Linear model results when investigating whether species explains the variance in the HS:HO ratio. Appendix S10: Linear model results when investigating whether an interaction between species and mating system (fixed effects) explains the variance in the rate of seed failure. Appendix S11: Comparisons of capsule production with and without seeds in the hand outcrossed (HO), hand selfed (HS), and autonomously selfed (AS) mating systems for P. hirsutus and P. smallii . Appendix S12: Linear model results when investigating whether species explains the variance in the AS:HS ratio. Appendix S13: Hourly visitation rate for each plant species per observation day. Appendix S14: Hourly visitation rate per day by bees differs across focal plant populations. [file ECE3-16-e73692-s001.docx]

**Appendix 1:** Number of *P. hirsutus* and *P. smallii* plants used for the overall mating system study and for specific treatment group comparisons.

| **Plant species** | **Total number of plants** | **Plants that produced HO, HS and AS seeds** | **Plants that produced both HO and HS seeds** | **Plants that produced both HS and AS seeds** |
| --- | --- | --- | --- | --- |
| *P. hirsutus* | 11 | 4 | 5 | 4 |
| *P. smallii* | 8 | 5 | 5 | 5 |

**Appendix 2:** Linear model results when investigating whether flower shape explains the variation in pollinator thorax height.

Model: Poll_model1 <- lm(pollinator_height ~ flower_shape, data = bee_data2022_nona)

|  | Estimate | Std. Error | t value | Pr(>\|t\|) |
| --- | --- | --- | --- | --- |
| Intercept | 2.9000 | 0.2635 | 11.006 | <2e-16 *** |
| flower_shapepersonate | 0.6576 | 0.3999 | 1.644 | 0.102 |
| flower_shapetubular | 0.2131 | 0.3120 | 0.683 | 0.495 |

**Appendix 3:** Two sample t-test used to investigate whether species explains the variance in pollen:ovule ratios.

t.test(OvuleCount ~ Species, data = Ovule_summ, paired = FALSE, conf.level = 0.95)

| t | df | p-value | 95% confidence interval (low) | 95% confidence interval (high) | *P. hirsutus* mean | *P. smallii* mean |
| --- | --- | --- | --- | --- | --- | --- |
| -2.1673 | 2.5463 | 0.1346 | -241.76889 | 57.78187 | 28.18959 | 120.18310 |

**Appendix 4:** Linear model results when investigating whether species explains the variance in ovule number.

t.test(OvuleCount ~ Species, data = Ovule_summ, paired = FALSE, conf.level = 0.95)

| t | df | p-value | 95% confidence interval (low) | 95% confidence interval (high) | *P. hirsutus* mean | *P. smallii* mean |
| --- | --- | --- | --- | --- | --- | --- |
| 3.1786 | 3.9946 | 0.03365 | 8.094582 | 120.349862 | 170.8889 | 106.6667 |

**Appendix 5:** Linear model results when investigating whether species explains the variance in pollen grains per flower.

t.test(WholeFlowerCount ~ Species, data = Pollen_summ, paired = FALSE, conf.level = 0.95)

| t | df | p-value | 95% confidence interval (low) | 95% confidence interval (high) | *P. hirsutus* mean | *P. smallii* mean |
| --- | --- | --- | --- | --- | --- | --- |
| -2.3367 | 3.9168 | 0.08106 | -15849.665 | 1429.221 | 4229.778 | 11440.000 |

**Appendix 6:** Hurdle model results when investigating the variance in seed production and number per capsule.

Model: HurdleModel <- glmmTMB(number_of_seeds ~ species * mating_system, ziformula = ~ species * mating_system, family = truncated_poisson(), data = Master_YNmatingsystemseeddata)

| AIC | BIC | logLik | - 2*log(L) | df. resid |
| --- | --- | --- | --- | --- |
| 3907.8 | 3947.4 | -1941.9 | 3883.8 | 188 |

Conditional model:

|  | Estimate | Std. Error | z value | Pr(>\|z\|) |
| --- | --- | --- | --- | --- |
| (Intercept) | 4.51117 | 0.01772 | 254.63 | **< 2e-16 ***** |
| speciessmallii | -1.15861 | 0.02905 | -39.88 | **< 2e-16 ***** |
| mating_systemhand_outcross | 0.69537 | 0.02712 | 25.64 | **< 2e-16 ***** |
| mating_systemhand_self | 0.41487 | 0.03031 | 13.69 | **< 2e-16 ***** |
| speciessmallii:mating_systemhand_outcross | -0.19451 | 0.05382 | -3.61 | **0.000302 ***** |
| speciessmallii:mating_systemhand_self | -0.10782 | 0.05856 | -1.84 | 0.065575 . |

Zero-inflation model:

|  | Estimate | Std. Error | z value | Pr(>\|z\|) |
| --- | --- | --- | --- | --- |
| (Intercept) | -2.4567 | 0.6016 | -4.084 | **4.43e-05 ***** |
| speciessmallii | 1.9047 | 0.6351 | 2.999 | **0.00271 **** |
| mating_systemhand_outcross | -15.5575 | 2263.4518 | -0.007 | 0.99452 |
| mating_systemhand_self | -19.5870 | 17666.0975 | -0.001 | 0.99912 |
| speciessmallii:mating_systemhand_outcross | 13.5446 | 2263.4520 | 0.006 | 0.99523 |
| speciessmallii:mating_systemhand_self | 19.3658 | 17666.0976 | 0.001 | 0.99913 |

**Appendix 7:** Estimated Marginal Means compared between treatment groups for both species’ seed number.

Model: mixedmod1 <- glmer(number_of_seeds ~ species * mating_system + (1 | family_code), data = Master_matingsystemseeddata, family=poisson())

| contrast | estimate | SE | df | z ratio | p value |
| --- | --- | --- | --- | --- | --- |
| *P. hirsutus* | | | | | |
| auto_self - hand_outcross | -0.316 | 0.0347 | Inf | -9.114 | **<0.0001** |
| auto_self - hand_self | -0.150 | 0.0343 | Inf | -4.388 | **<0.0001** |
| hand_outcross - hand_self | 0.166 | 0.0340 | Inf | 4.889 | **<0.0001** |
| *P. smallii* | | | | | |
| auto_self - hand_outcross | -0.767 | 0.0527 | Inf | -14.549 | **<0.0001** |
| auto_self - hand_self | -0.536 | 0.0527 | Inf | -10.161 | **<0.0001** |
| hand_outcross - hand_self | 0.231 | 0.0620 | Inf | 3.731 | **0.0006** |

**Appendix 8:** Linear model results when investigating whether an interaction between species and mating system (fixed effects) explains the variance in seed mass.

Model: mixedmod2 <- lmer(indiv_seedmass ~ species * mating_system + (1 | family_code), data = seedweightdata)

Random effects:

| Groups | Name | Variance | Std. Dev. |
| --- | --- | --- | --- |
| Family_code | (Intercept) | 0.0010195 | 0.03193 |
| Residual |  | 0.0006753 | 0.02599 |

Fixed effects:

|  | Estimate | Std. Error | df | t value | Pr(>\|t\|) |
| --- | --- | --- | --- | --- | --- |
| (Intercept) | 0.134258 | 0.014822 | 30.618298 | 9.058 | 3.61e-10 *** |
| speciessmallii | 0.024757 | 0.018355 | 25.037890 | 1.349 | 0.189 |
| mating_systemhand_self | 0.004795 | 0.016148 | 17.922674 | 0.297 | 0.770 |
| mating_systemauto_self | -0.005155 | 0.015617 | 22.835546 | -0.330 | 0.744 |
| speciessmallii:mating_systemhand_self | 0.025578 | 0.022737 | 17.631335 | 1.125 | 0.276 |
| speciessmallii:mating_systemauto_self | 0.027358 | 0.022218 | 21.356150 | 1.231 | 0.232 |

**Appendix 9:** Linear model results when investigating whether species explains the variance in the HS:HO ratio.

Model: mixedmod3.3 <- lm(HS_HO_Comp_per_fam_from_standardized_seedvalues ~ species, data = matingsystemHSHOseeds)

Fixed effects:

|  | Estimate | Std. Error | t value | Pr(>\|t\|) |
| --- | --- | --- | --- | --- |
| (Intercept) | 0.8547 | 0.1023 | 8.355 | 3.19e-05 *** |
| speciessmallii | -0.1310 | 0.1447 | -0.906 | 0.392 |

**Appendix 10:** Linear model results when investigating whether an interaction between species and mating system (fixed effects) explains the variance in the rate of seed failure.

Model: mixedmod6.1 <- lmer(failure_ratio_per_fam_trtmt_combo ~ species * mating_system + (1 | family_code), data = FailureRatio)

Random effects:

| Groups | Name | Variance | Std. Dev. |
| --- | --- | --- | --- |
| Family_code | (Intercept) | 6.946e-05 | 0.008334 |
| Residual |  | 3.221e+00 | 1.794758 |

Fixed effects:

|  | Estimate | Std. Error | df | t value | Pr(>\|t\|) |
| --- | --- | --- | --- | --- | --- |
| (Intercept) | 0.06568 | 0.59826 | 33.00000 | 0.110 | 0.9132 |
| speciessmallii | 2.38620 | 0.87210 | 28.70104 | 2.736 | 0.0105 * |
| mating_systemhand_outcross | -0.06566 | 0.94593 | 27.67543 | -0.069 | 0.9452 |
| mating_systemhand_self | -0.06566 | 1.00107 | 27.03004 | -0.066 | 0.9482 |
| speciessmallii:mating_systemhand_outcross | -2.30290 | 1.35436 | 25.65830 | -1.700 | 0.1012 |
| speciessmallii:mating_systemhand_self | -1.66624 | 1.43144 | 25.20852 | -1.164 | 0.2553 |

Estimated marginal means:

| Contrast | estimate | SE | df | t ratio | p value |
| --- | --- | --- | --- | --- | --- |
| **Mating system = Autonomously Selfed (AS)** | | | | | |
| *P. hirsutus* – *P. smallii* | -2.3862 | 0.885 | 30.0 | -2.696 | **0.0114** |
| **Mating system = Hand Outcrossed (HO)** | | | | | |
| *P. hirsutus* – *P. smallii* | -0.0833 | 1.060 | 32.6 | -0.078 | 0.9381 |
| **Mating system = Hand Selfed (HS)** | | | | | |
| *P. hirsutus* – *P. smallii* | -0.7200 | 1.170 | 32.4 | -0.615 | 0.5431 |

**Appendix 11** Comparisons of capsule production with and without seeds in the hand outcrossed (HO), hand selfed (HS), and autonomously selfed (AS) mating systems for *P. hirsutus* and *P. smallii*. Green areas of bars represent flowers that produced seeds and orange areas represent flowers that failed to produce seeds.

**Appendix 12:** Linear model results when investigating whether species explains the variance in the AS:HS ratio.

Model: mixedmod4.4 <- lm(AS_HS_Comp_per_fam_from_standardized_seedvalues ~ species, data = matingsystemASHSseeds)

|  | Estimate | Std. Error | t value | Pr(>\|t\|) |
| --- | --- | --- | --- | --- |
| (Intercept) | 0.80811 | 0.24308 | 3.324 | 0.0127 * |
| speciessmallii | 0.04219 | 0.32613 | 0.129 | 0.9007 |

**Appendix 13:** Hourly visitation rate for each plant species per observation day. Population location reflects the nearest town to each plant population used for pollinator observations. All pollinator observations were conducted on separate days except *P. calycosus* and *P. hirsutus* which co-occurred at a single field site in Clarksville, Tennessee (USA).

| **Plant species** | **Flower shape** | **Population location** | **Observation day per population** | **Hourly visitation rate** |
| --- | --- | --- | --- | --- |
| *P. australis* | Tubular | Congaree, SC | 1 | 6.5 |
| *P. australis* | Tubular | Congaree, SC | 2 | 6.666 |
| *P. australis* | Tubular | Congaree, SC | 3 | 13 |
| *P. australis* | Tubular | Congaree, SC | 4 | 5 |
| *P. australis* | Tubular | McBee, SC | 1 | 7 |
| *P. canescens* | Tubular | Andrews, NC | 1 | 18 |
| *P. canescens* | Tubular | Andrews, NC | 2 | 13.333 |
| *P. calycosus* | Open | Clarksville, TN | 1 | 1 |
| *P. calycosus* | Open | Clarksville, TN | 2 | 3.333 |
| *P. smallii* | Open | Linville, NC | 1 | 9.5 |
| *P. smallii* | Open | Linville, NC | 2 | 5.5 |
| *P. hirsutus* | Personate | Clarksville, TN | 1 | 0.333 |
| *P. hirsutus* | Personate | Clarksville, TN | 2 | 0.333 |
| *P. oklahomensis* | Personate | Norman, OK | 1 | 2 |
| *P. oklahomensis* | Personate | Norman, OK | 2 | 4 |
| *P. tenuiflorus* | Personate | Aurora, KY | 1 | 4 |

**Appendix 14** Hourly visitation rate per day by bees differs across plant populations. (A) Visitation rate for each focal *Penstemon* subsect. *Penstemon* species. (B) Visitation rate averaged for each flower morphology. Colored points represent the hourly visitation rate for each day spent observing pollinators. The fill color of each point in panel A matches flower shape (brown: tubular, purple: open-tubed, yellow: personate), while grey points with a red outline indicate the mean visitation rate for each species (panel A) and each flower morphology (panel B). The red asterisk near species’ names in panel A corresponds with the flowers shown under panel B. Box lines (panel B) indicate the median hourly visitation rate for each flower morphology.
